# Nursing Services of the Crown

**Published:** 1918-11-16

**Authors:** 


### NURSING SERVICES OF THE CROWN.

### The Territorial Force Nursing Service (Miss Sidney Browne, R.R.C., Matron-in-Chief).

LAST week we dealt with the Military Nursing Services of the Crown, but our notice was necessarily incomplete as we had not then the figures of the above Service, which we have now the pleasure to give. We gave the number of nurses at the present time in the Q.A.I.M.N.S. and Reserve as approximately 8,000, in addition to which there are some 6,000 V.A.D. Nursing Members. The total number of trained nurses enrolled by the Territorial Force Nursing Service, who have served in Military and Territorial Hospitals since the outbreak of war, is 7,269, and the V.A.D. Nursing Members and Special Military Probationers in this Service have been 5,200. At the present time the number of trained nurses serving under the Territorial Force amounts to 5,147, of whom 2,000 are working abroad in Territorial and Regular Military Hospitals, in Casualty Clearing Stations, Hospital Trains and Ships. The V.A.D. Nursing Members working under the Territorial Force Nursing Service in hospitals at home number 3,643, whilst 1,485 are at present employed abroad. At the commencement of the war 3,000 trained nurses were called up within a week and constituted the original staff of the Territorial Force Nursing Service.

The efficiency of the Service is shown by the facts that 11 Military Medals have been won and a large number of members have received the Royal Red Cross. The devotion and bravery of these nurses, and the risks attaching to service, are demonstrated by the circumstance that 38 of these noble women have lost their lives in the war.

### Voluntary Aid and Devonshire House (Lady Ampthill, Commandant-in-Chief).

IN this, the Senior Women's Service, there were originally 80,000 members, *i.e.* at the outbreak of the war. This number represented all who had enrolled in connection with the county and other organisations in accordance with the V.A.D. scheme which was inaugurated in August 1909. The Secretary of State for War issued on August 16, 1909, to the Territorial Force County Associations in England and Wales a scheme for the organisation of voluntary aid for sick and wounded in the event of war in the *home territory*. The V.A.D. organisation is a part of the Joint Committee of the British Red Cross Society and the Order of St. John of Jerusalem.

The V.A.D.s may be classified as follows:

(1) Nursing Members. These work in Naval, Military, Territorial, and American Hospitals at home and abroad.

(2) Members (unpaid) working in Auxiliary (B.R.C.S.) Hospitals at home.

(3) General Service Members working in Naval, Military, Territorial, and American Hospitals in England, France, Salonika, and Italy.

(4) Joint Committee Members, who staff eight hospitals in France. They are both Nursing and General Service Members.

Lady Ampthill, Commandant-in-Chief of the V.A.D. organisation has, according to the latest information available, employed in the V.A.D. Service about 120,000 women. In August 1914 the V.A.D. Members numbered about 80,000, but there are no complete returns. The first batch of V.A.D. Nursing Members were posted to Military Hospitals in March 1915, and the first batch of V.A.D. General Service Members commenced work in August 1915. There are at the present time approximately 12,000 V.A.D. Nursing Members in Military Hospitals, 60,000 Members in the Auxiliary Hospitals, and 6,000 V.A.D. Members engaged in General Service. This leaves 42,000 V.A.D. workers to classify.

H.R.H. Princess Mary has worked for a considerable time as a V.A.D. at the headquarters at Devonshire House, and is devoted to her duties. Among the many pleasant and notable incidents connected with the assembly of thousands of people, representing all classes, at Buckingham Palace on Monday last, not the least interesting and impressive was a large detachment of V.A.D.s, who marched down from Devonshire House to Buckingham Palace in mass formation, and when they modestly took up one of the least prominent positions were requested to come up higher. This speaks extraordinarily well for the high tone, modesty, and organisation of the V.A.D. Force, thanks largely to the wonderful powers which Lady Ampthill has brought to bear upon this organisation since she accepted the office of Commandant-in-Chief. This organisation has attracted many able administrators, and amongst them none is more efficient or holds a higher place than Lady Oliver, who is second in command of the V.A.D.s organisation at Devonshire House.

We gave a full account of the nursing activities of the Joint War Committee of the British Red Cross and the Order of St. John, of which Miss Swift is the able matron-in-chief, on page 117 of last week's THE HOSPITAL.

(*Concluded from page* 117, *November* 9.)

